# Tiotropium inhibits proinflammatory microparticle generation by human bronchial and endothelial cells

**DOI:** 10.1038/s41598-019-48129-w

**Published:** 2019-08-12

**Authors:** Tommaso Neri, Valentina Scalise, Ilaria Passalacqua, Chiara Sanguinetti, Stefania Lombardi, Laura Pergoli, Valentina Bollati, Roberto Pedrinelli, Pierluigi Paggiaro, Alessandro Celi

**Affiliations:** 1 Centro Dipartimentale di Biologia Cellulare Cardiorespiratoria, Dipartimento di Patologia Chirurgica, Medica, Molecolare e di Area Critica, Università degli Studi di Pisa, and Dipartimento Cardiotoracovascolare, Azienda Ospedaliero-Universitaria Pisana, Pisa, Italy; 2 SSD Analisi ChimicoCliniche, Ospedale Apuane Toscana Nordovest, Massa, Italy; 3 EPIGET – Epidemiology, Epigenetics and Toxicology Lab, Department of Clinical Sciences and Community Health, Università degli Studi di Milano, Milan, Italy

**Keywords:** Translational research, Cell signalling

## Abstract

Tiotropium is a muscarinic antagonist that reduces the risk of acute exacerbations of chronic obstructive pulmonary disease, possibly through an as yet incompletely characterized anti-inflammatory activity. We hypothesized that muscarinic activation of bronchial epithelial cells and endothelial cells causes the release of proinflammatory microparticles and that tiotropium inhibits the phenomenon. Microparticle generation was assessed by a functional assay, by flow cytometry and by NanoSight technology. Immortalized bronchial epithelial cells (16HBE) and umbilical vein endothelial cells were treated with acetylcholine in the presence of varying concentrations of tiotropium. Intracellular calcium concentration, extracellular regulated kinase phosphorylation and chemokine content in the conditioned media were assessed by commercial kits. Acetylcholine causes microparticle generation that is completely inhibited by tiotropium (50 pM). Microparticles generated by acetylcholine-stimulated cells increase the synthesis of proinflammatory mediators in an autocrine fashion. Acetylcholine-induced upregulation of microparticle generation is inhibited by an inhibitor of extracellular regulated kinase phosphorylation and by a phospholipase C inhibitor. Tiotropium blocks both extracellular regulated kinase phosphorylation and calcium mobilization, consistent with the hypothesis that the drug prevents microparticle generation through inhibition of these critical pathways. These results might contribute to explain the effect of tiotropium in reducing acute exacerbations of chronic obstructive pulmonary disease.

## Introduction

Tiotropium is a long acting muscarinic antagonist (LAMA) routinely used as maintenance therapy in chronic obstructive pulmonary disease (COPD)^1^ and also approved for severe asthma^2^. Tiotropium causes symptom relief, an activity attributed to its bronchodilating properties. However, it has also been demonstrated that this drug reduces the risk of acute exacerbations of COPD (AECOPD) compared to the long acting β_2_-agonist, salmeterol^3^. The mechanisms whereby tiotropium confers a protection against AECOPD, that represent transient inflammatory events, has been the matter of debate. While bronchodilation is likely involved, other potential mechanisms, including anti-inflammatory effects, have been postulated^4^.

Cells generate extracellular vesicles (EV) under different conditions, including activation and apoptosis. EV vary in size and composition depending on the cell type and the stimulus used for their generation. The nomenclature of EV is still being refined and it is commonly deemed extremely difficult to define a specific type of EV solely based on size, composition and/or biogenesis^5^. For consistency with previous publications, throughout this paper we will use the somewhat arbitrary term microparticles (MP) to designate vesicles ranging in size between approximately 50 and 1000 nm and expressing negatively charged phospholipids on the outer membrane. According to the Minimal Information for Studies on Extracellular Vesicles 2018 position statement^5^, MP as defined above would fall under the definition of both small (<200 nm) and medium-large (>200 nm) EV.

EV have been implicated in a variety of physiological and pathological conditions^6^. Potentially relevant to pulmonary diseases, in *in vitro* experiments we have shown that MP generated by human mononuclear cells upon stimulation with different agonists induce the synthesis of proinflammatory mediators by human lung epithelial cells^7,8^. The effect is mediated by NF-κB activation^9^. Human studies have demonstrated an increased number of MP of endothelial origin in the peripheral blood and sputum of COPD patients, particularly during AECOPD^10–12^.

The mechanisms of MP generation are complex and only partially understood. We and others have demonstrated a role for intracellular calcium mobilization^8,13^. Furthermore, phosphorylation of extracellular signal-regulated kinases (ERK) has also been linked to MP formation^14,15^.

Based on the known role of muscarinic activation in both the modulation of intracellular calcium concentration ([Ca^++^]_i_) and in ERK phosphorylation^16,17^, we hypothesized that muscarinic agonists induce the generation of proinflammatory MP by airway epithelial and endothelial cells, and that tiotropium inhibits this phenomenon.

## Results

### ACh stimulates MP generation from bronchial epithelial and endothelial cells

**Figure 1:**
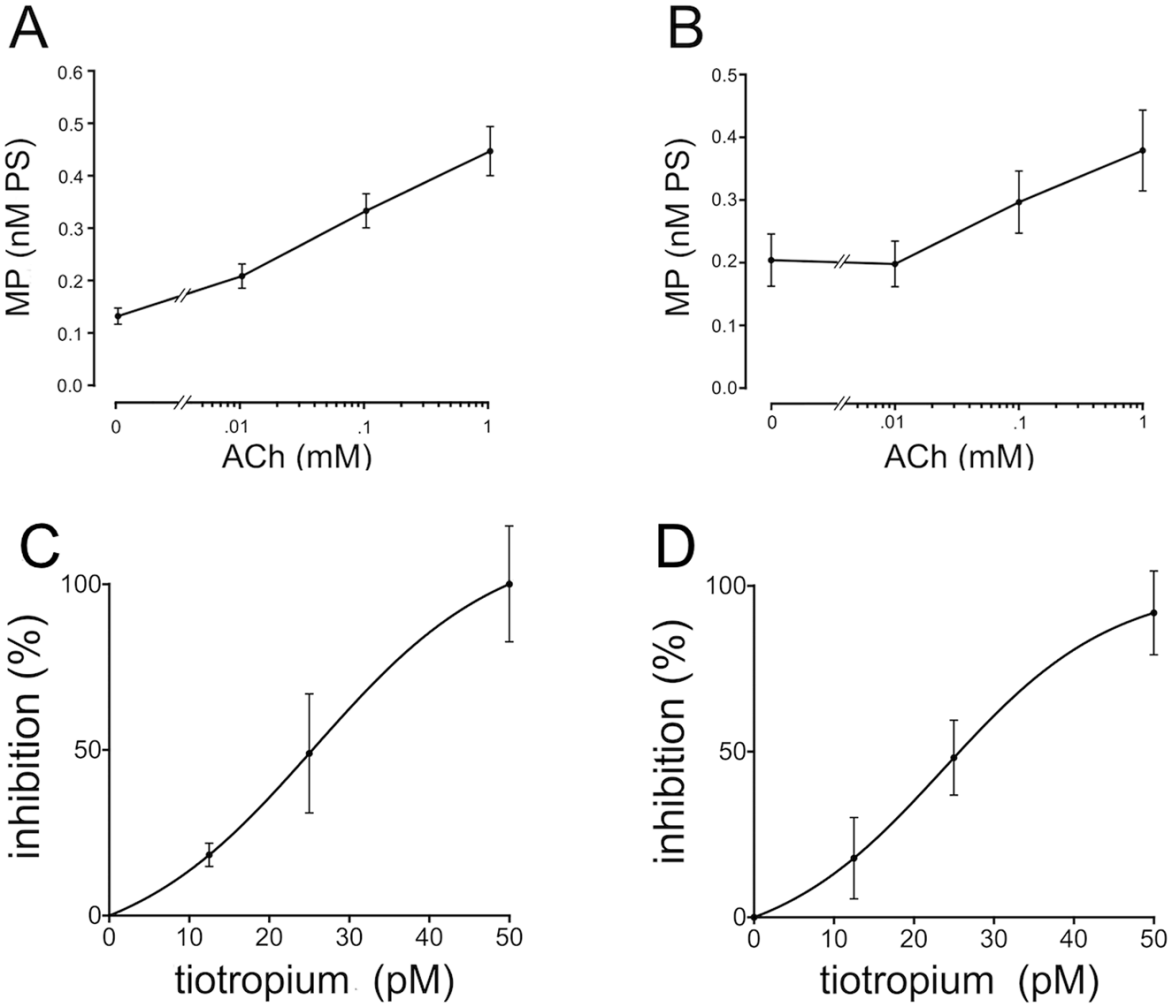
ACh stimulates and tiotropium inhibits MP generation. (**A**,**B**) dose response of the effect of ACh stimulation for 16HBE (**A**) and HUVEC (**B**). (**C**,**D**) dose-response of the effect of tiotropium on ACh-induced generation of MP by 16HBE cells (**C**) and HUVEC (**D**). Data are mean ± SEM from 3 consecutive experiments.

**Figure 2:**
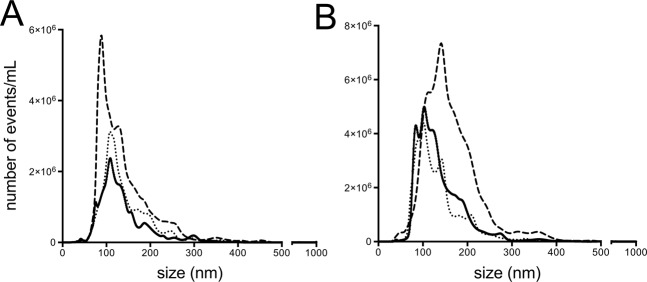
NanoSight analysis of MP generation by 16HBE cells (**A**) and HUVEC (**B**) under different experimental conditions. Solid line: baseline; dashed line: ACh-stimulated cells; dotted line: ACh-stimulated cells in the presence of tiotropium (50 pM). Data from 1 experiment representative of 2.

**Figure 3:**
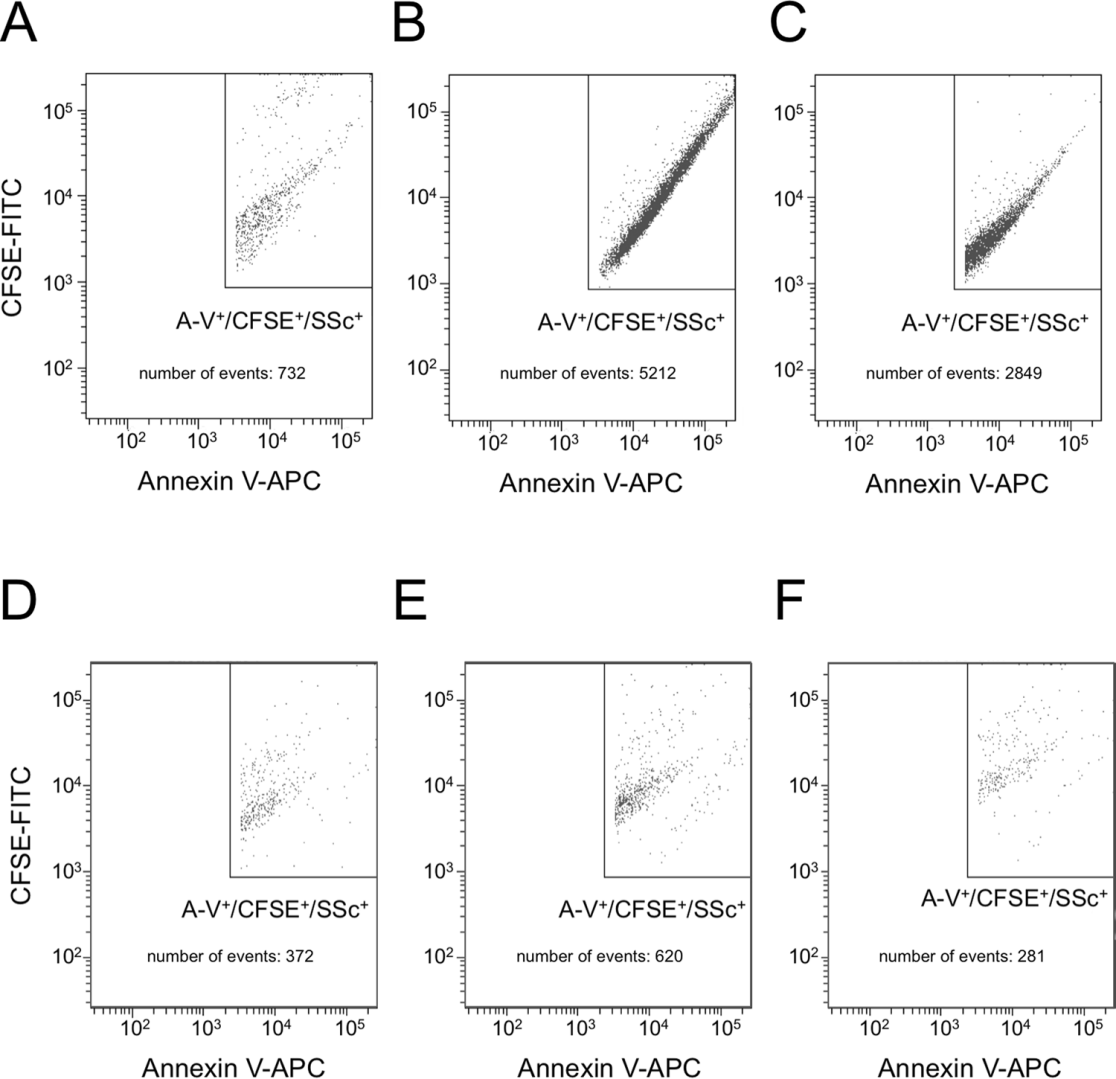
Flow cytometry analysis of MP generated by 16HBE cells (**A**–**C**) and HUVEC (**D**–**F**). Baseline (**A**,**D**) ACh-treated (**B**,**E**) ACh-treated in the presence of tiotropium, 50 pM (**D**,**F**). Data from 1 experiment representative of 3.

First, we tested the hypothesis that muscarinic stimulation upregulates the release of MP by the immortalized bronchial epithelial cells (16HBE) and human umbilical vein endothelial cells (HUVEC). ACh stimulation (1 h) caused a dose-dependent increase in MP generation, as assessed by the Zymuphen assay that measures phosphatidylserine (PS) content in the conditioned medium (Fig. 1A,B). To better define the PS-containing vesicles generated under these experimental conditions, both the NanoSight technology^18^, and flow cytometry were also used (Figs 2 and 3, respectively).

### Tiotropium inhibits ACh-induced generation of MP

Using the same cell model and the Zymuphen assay, we then tested the effect of tiotropium in ACh-induced MP generation. As shown in Fig. 1C,D, preincubation with tiotropium inhibited the effect of ACh (1 mM) in both cell types. Inhibition reached 100% at a concentration of 50 pM.

### NanoSight and flow cytometry analysis of MP generation

Besides the presence of negatively charged phospholipids on the external membrane, assessed by the Zymuphen assay, MP are also defined by size (50–1000 nm) and by the presence of intact cytoplasm. To better characterize the EV generated in our experimental conditions we therefore repeated some of the previously described experiments using NanoSight and flow cytometry.

In baseline conditions, 16HBE cells and HUVEC generate particles in the size range of MP. ACh caused an approximately 2.5-fold and 1.9-fold increase of the area under the curve for 16HBE cells and HUVEC, respectively; tiotropium (50 pM) inhibited the effect by 78% and >100%, respectively (Fig. 2).

Figure 3 reports the results obtained by flow cytometry. Again, the number of events falling under our definition for MP, i.e. within the FSc/SSc range of the 0.9 µm beads, positive for annexin-V and therefore expressing negatively charged phospholipids, and positive for CFSE and therefore representing closed vesicles enclosing cytoplasmic molecules, are increased in both cell types by ACh stimulation (1 mM) by 212% and 414%, respectively; on average, tiotropium inhibits the effect almost completely.

### Role of calcium mobilization

**Figure 4:**
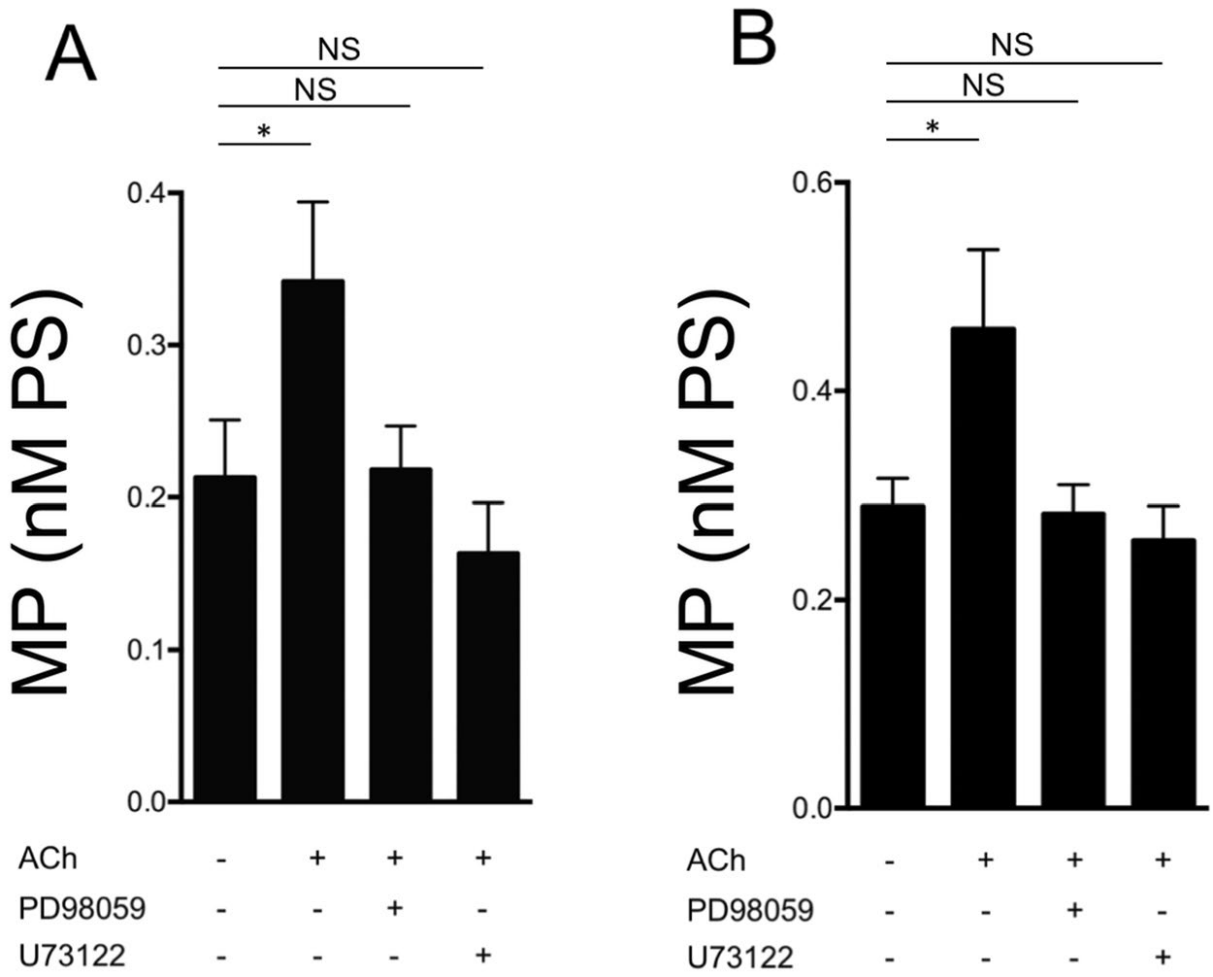
Inhibition of ERK phosphorylation and of phospholipase C reduces ACh-induced MP synthesis. Effects of the inhibitor of ERK phosphorylation, PD98059, (1 μM) and of the phospholipase-C inhibitor, U73122, (1 μM) on MP generation by ACh-stimulated 16HBE cells (**A**) and HUVEC (**B**). Data from 4 consecutive, independent experiments; ANOVA for repeated measures followed by Fisher’s least significant difference test. *p < 0.05 for ACh-stimulated vs. unstimulated cells. NS: Not significant.

The role of calcium mobilization on the generation of EV has been previously reported^8,13^. We confirmed that inhibition of calcium mobilization with the phspholipase-C inhibitor, U73122, completely inhibited MP generation in ACh-stimulated 16HBE cells and HUVEC (Fig. 4).

**Figure 5:**
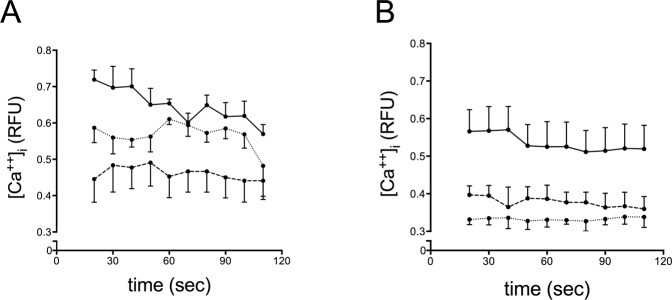
Modulation of [Ca^++^]_i_ by ACh and tiotropium in 16HBE cells (**A**) and HUVEC (**B**). Dashed line: baseline; solid line: ACh-stimulated cells; dotted line: ACh-stimulated cells in the presence of tiotropium. Data are mean ± SEM from 3 consecutive experiments.

Tiotropium reduced [Ca^++^]_i_ in both 16HBE cells and HUVEC stimulated with ACh (1 mM), lending support to the hypothesis that the inhibition of MP generation in our experimental conditions is, at least in part, mediated through a reduction of [Ca^++^]_i_ (Fig. 5).

**Figure 6:**
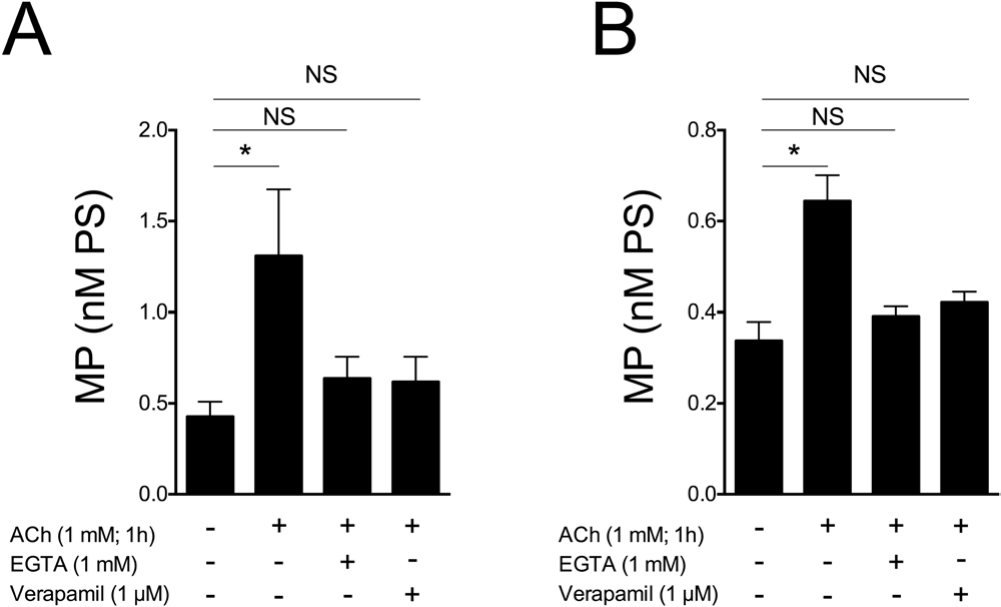
Inhibition of MP generation by EGTA and verapamil in 16HBE (**A**) and HUVEC (**B**). Data from 3 independent consecutive experiments. ANOVA for repeated measures followed by Fisher’s least significant difference test. *p < 0.05 for ACh-stimulated vs. unstimulaed cells. NS: not significant.

To better understand the contribution of extracellular calcium in MP generation, we used an L-type calcium channel inhibitor (verapamil, 1 μM, 30 min. of preincubation) and an extracellular calcium ion chelator [Ethylene glycol-bis(2-aminoethylether)-*N,N,N*′*,N*′-tetraacetic acid, EGTA, 1 mM; 30 min. of preincubation]. Both substances inhibit, almost completely, the generation of MP. These data show that both calcium released from the endoplasmic reticulum and the influx of calcium from the outside contribute to the liberation of MP (Fig. 6).

### Role of ERK phosphorylation

**Figure 7:**
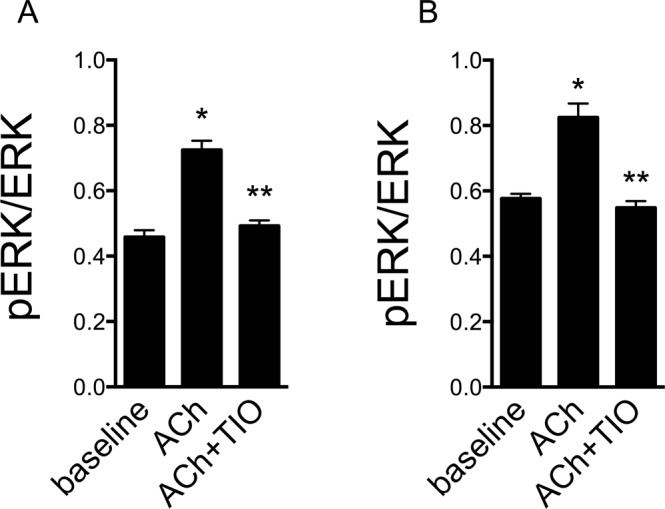
ACh stimulates and tiotropium inhibits ERK phosphorylation. pERK/ERK ratio in 16HBE cells (**A**) and HUVEC (**B**) in the absence and in the presence of ACh and tiotropium (TIO). Data form 4 independent consecutive experiments. ANOVA for repeated measures followed by Fisher’s least significant difference test. *p < 0.05 for ACh-stimulated vs. unstimulated cells; **p < 0.05 for ACh-stimulated cells in the presence of tiotropium vs. cells stimulated in the absence of tiotropioum.

Kinase phosphorylation is another well-established mechanism of EV generation. As shown in Fig. 4, PD98059, a MEK inhibitor that prevents ERK phosphorylation, abolished the effect of ACh (1 mM) on MP generation. Figure 7 shows the effects of ACh and tiotropium in ERK phosphorylation. These results are consistent with a role for muscarinic agonists and antagonists in ERK-mediated generation of MP.

### Proinflammatory effects of ACh-generated MP

**Figure 8:**
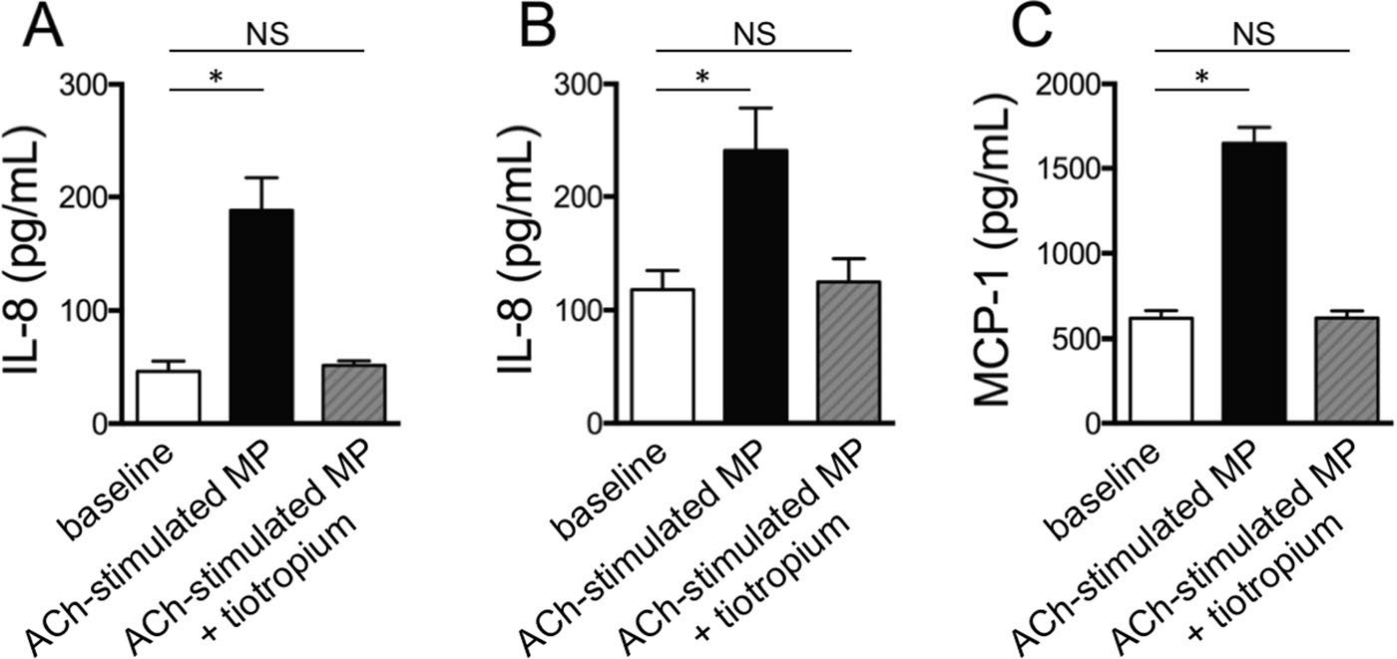
MP generated by ACh-stimulated cells are proinflammatory. Cytokine synthesis by 16HBE cells (**A**) and HUVEC (**B**,**C**) in baseline conditions (open bars), upon incubation with MP generated by ACh-stimulated cells (solid bars) and upon incubation with MP generated by ACh-stimulated cells in the presence of tiotropium (50 pm) (hatched bars). Data from 3 consecutive independent experiments. ANOVA followed by Fisher least significant difference test. *p < 0.05.

We and others have previously shown that MP generated by different cell types and upon stimulation with different agonists upregulate the synthesis of proinflammatory mediators by target cells^7–9,19,20^. Here, we confirm that ACh-induced MP generated in both 16HBE cells and HUVEC upregulate the autocrine synthesis of IL-8 and, limited to HUVEC, of MCP-1 as assessed by ELISA on the conditioned medium of unstimulated and MP-stimulated cells. As we have repeatedly observed that bronchial epithelial cells do not respond to MP with an increased synthesis of MCP-1^7^ (and unpublished observations), we did not perform the relevant experiments in the current setting. As expected, the conditioned medium of ACh-stimulated cells pretreated with tiotropium, that contains approximately the same amount of MP as the conditioned medium of unstimulated cells, did not upregulate cytokine synthesis (Fig. 8).

**Figure 9:**
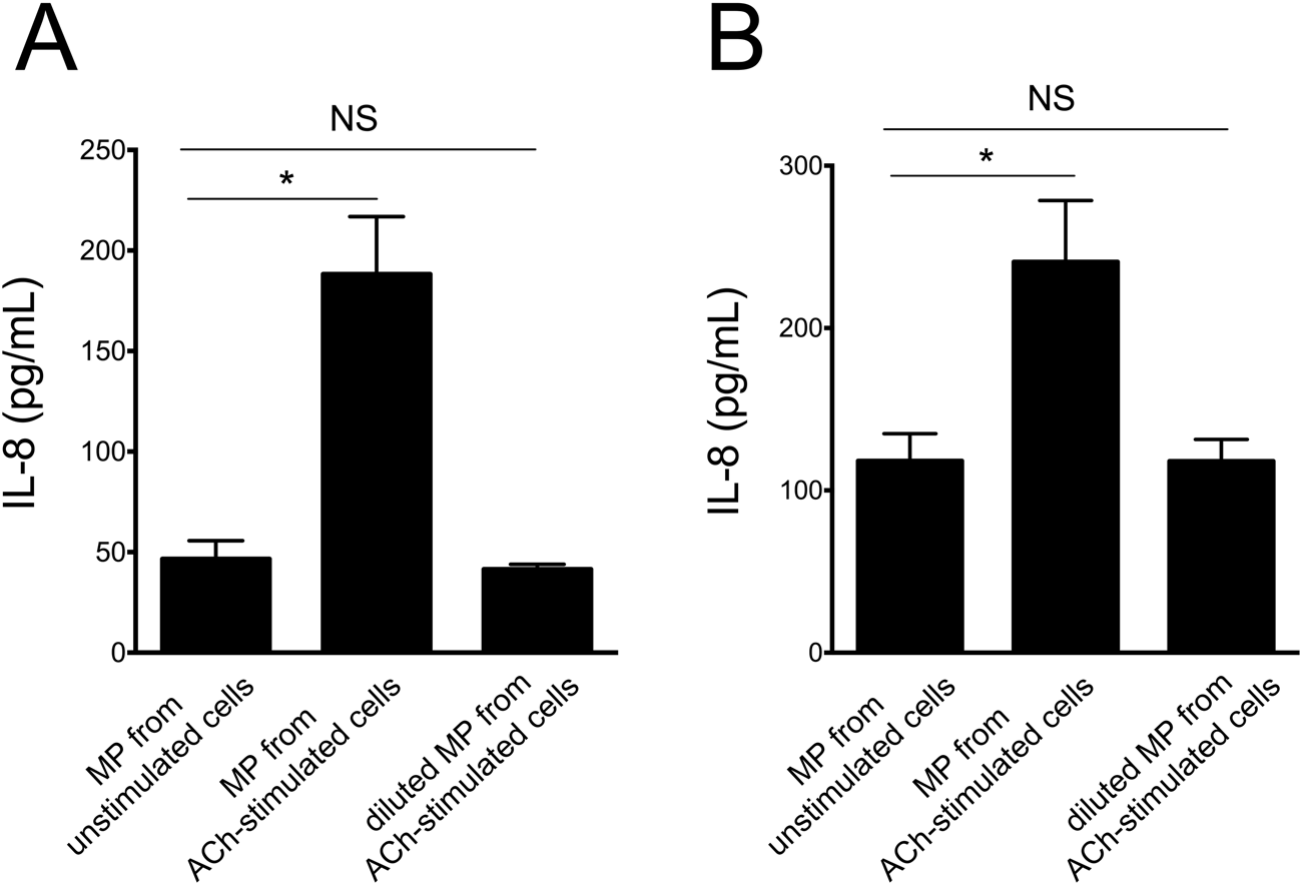
IL-8 synthesis by 16HBE (**A**) and HUVEC (**B**) upon incubation with MP from unstimulated cells, undiluted MP form ACh-stimulated cells, and MP from ACh stimulated cells after dilution to match the number of MP from unstimulated cells. Data from 3 consecutive independent experiments. ANOVA followed by Fisher least significant difference test. *p < 0.05 NS: Not significant.

To confirm that total MP number, rather than composition, was responsible for the different IL-8 stimulation by ACh-induced and baseline MP, we incubated 16HBE and HUVEC with ACh-induced MP diluted to match the concentration of MP contained in the supernatant of unstimulated cells. The results show that diluted ACh-induced MP do not stimulate IL-8 synthesis (Fig. 9).

### Effects of ipratropium

**Figure 10:**
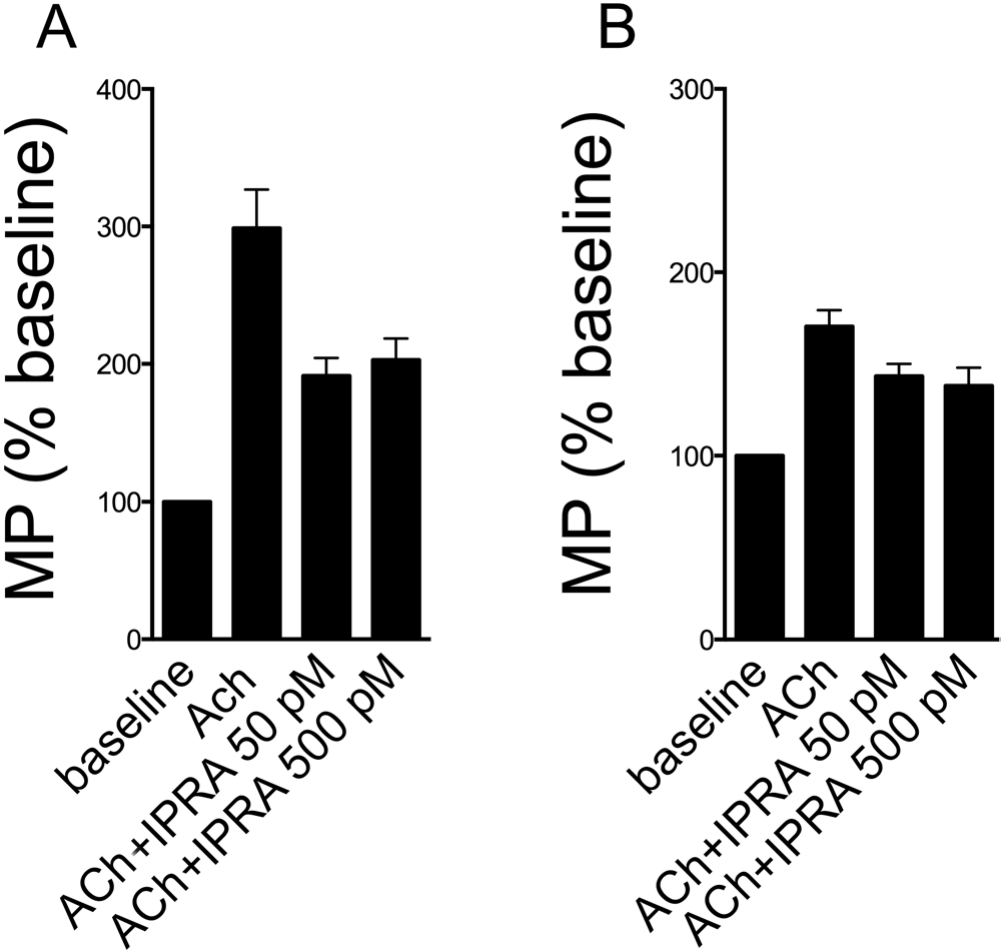
Inhibition of MP generation by 16HBE (**A**) and HUVEC (**B**) by ipratropium. Data form 4 consecutive experiments.

Ipratropium is a short acting muscarinic antagonist still used in clinical practice. We investigated whether the inhibitory effects described above are shared by this molecule. Ipratropium at the concentration of 50 pM caused an approximately 45% inhibition in both cell lines; increasing the concentration up to 10 fold did not further increase the inhibitory effect (Fig. 10).

## Discussion

Our data demonstrate an hitherto unrecognized effect of tiotropium that might contribute to explain its therapeutic effect, i.e. the inhibition of the generation of MP by human bronchial and endothelial cells after muscarinic stimulation.

The analysis of EV is complex and a standard approach for their definition, identification and enumeration is still lacking^5^. We performed the critical experiments using three independent approaches (i.e. PS analysis, flow cytometry and NanoSight technology) to increase the reliability of the results.

Muscarinic activation has long been recognized as a factor potentially involved in airway inflammation. Stimulation with ACh, the prototypical muscarinic agonist, causes the release of chemotactic activity by alveolar macrophages^21^. Profita *et al*. have shown an increase in IL-8 synthesis by human bronchial epithelial cells stimulated with ACh through an ERK-dependent mechanism^17^. Here, we show that ACh induces MP generation by human bronchial and endothelial cells. The effect is mediated, at least in part, through ERK phosphorylation as demonstrated by the effects of the inhibitor of ERK phosphorylation, PD98059. ACh-induced generation of MP is also mediated through an increase in [Ca^++^]_i_, as demonstrated by the complete inhibition obtained with the phospholipase C inhibitor, U73122, with the L-type calcium channel inhibitor, verapamil, and with the extracellular calcium ion chelator, EGTA. These data suggest that both extracellular calcium entry and release from intracellular stores might be sufficient to reach the necessary calcium concentration required for MP generation.

We then investigated the effects of tiotropium, a muscarinic antagonist used in clinical practice in patients with COPD and severe asthma, in the above mentioned phenomena. Tiotropium, at physiologically relevant concentrations in the low pM range, inhibits ERK phosphorylation, lowers [Ca^++^]_i_ and, ultimately, inhibits MP generation by ACh-stimulated bronchial and endothelial cells.

MP, originally identified through their procoagulant potential mediated by PS, are now recognized as critical components in numerous physiological and pathophysiological phenomena, including lung inflammation. *In vitro*, MP originated from human monocytes/macrophages activate NF-κB^9^ and upregulate the synthesis of chemokines by pulmonary epithelial cells^7,8^. In the current study we confirm that MP generated by ACh-stimulated bronchial and endothelial cells exert the same proinflammatory effect in an autocrine fashion. Preliminary data using cell-based *in vitro* models, *ex vivo* murine models and *in* vivo preclinical models have shown that bacterial infection causes the release of EV^22^. Finally, the number of E-selectin bearing MP of endothelial origin is increased during AECOPD^10^. These data are consistent with a model whereby MP are involved in the pathogenesis of COPD, most notably during AE.

**Figure 11:**
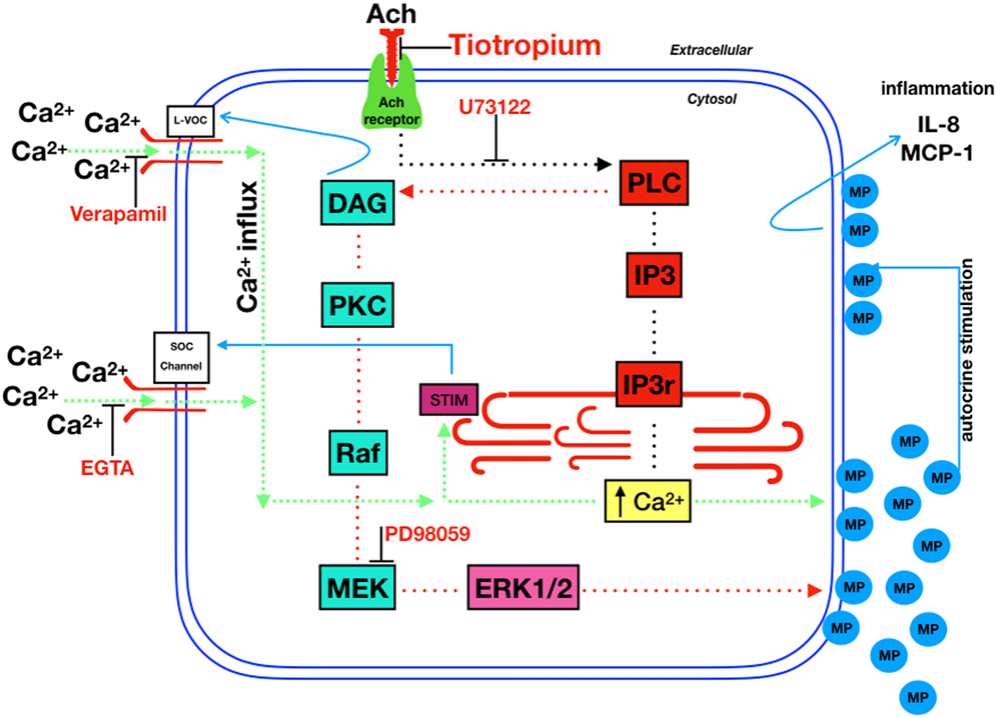
A graphical model proposed on the basis of the current results.

Figure 11 describes a model based on the current results.

A role for tiotropium in preventing lung inflammation has been previously postulated based on its clinical effects in the prevention of AECOPD and in *in vitro* data^23^, although most results were obtained with concentrations in the nM range^17,24^. Tiotropium has also been shown to attenuate IL-13-induced goblet cell metaplasia of human airway epithelial cells^25^.

Powrie *et al*. have measured sputum and serum inflammatory markers in patients treated with tiotropium and have found that while this drug reduced AECOPD by 52%, as expected, it did not affect inflammatory markers. The Authors concluded that tiotropium-induced prevention of AECOPD is not mediated by a reduction in airway or systemic inflammation^26^. It has been argued that sputum and serum markers might not truly reflect changes in bronchial mucosa^4^. However, it will be interesting to investigate the effect of tiotropium on MP concentration in relevant specimens from COPD patients.

In conclusion, we demonstrate that tiotropium, at physiologically relevant concentrations, inhibits the generation of MP by bronchial and endothelial cells. Preliminary data indicate that the short acting muscarinic antagonist, ipratropium, is less potent. A better understanding of alternative pharmacological effects of tiotropium and other muscarinic antagonists might prove instrumental in our constant effort to optimize COPD and asthma therapy.

## Methods

### Cell Culture

HUVEC, obtained from Life Technologies Italia (Monza, Italy), were maintained in M200 supplemented with 20% fetal bovine serum (FBS), low serum growth supplement (LSGS) and 2 mM glutamine.

16HBE (American Type Culture Collection, CRL-2741) were kindly provided by Dr. M. Profita (National Research Council, Palermo, Italy). 16HBE cells were maintained in MEM supplemented with 10% (vol/vol) FBS, 0.2 ml/mL L-glutamine and 2.5 mM HEPES buffer as described^27^. Both cell lines were maintained in a humidified 95% air/5% CO_2_ atmosphere at 37 °C.

### MP generation and purification

HUVEC and 16HBE cells, grown in 96-well plates until 90% confluent, were washed twice with pre-warmed phosphate buffered saline prior to stimulation. For MP generation analysis, ACh was resuspended first in distilled water and then in cell medium and added at different concentrations. After 60 min at 37 °C the supernatants were recovered and cleared by centrifugation at 16,000 x g for 5 min at room temperature to remove dead cells and big cell fragments that might have detached during the stimulation.

For experiments conducted at NanoSight, supernatants were centrifuged at 1,000, 2,000, and 3,000 × g for 15 min at 4 °C, and the resulting pellets of cell debris were discarded. An aliquot of 3 ml was then ultracentrifuged (BeckmanCoulter Optima-MAX-XP) at 110000 × g for 2 h at 4 °C, to obtain an MP-rich pellet. The pellet was resuspended with 800 μl triple 0.10 μm pore size membrane-filtered PBS.

All the experiments were conducted in serum free medium.

### Cell treatments

To understand the involvement of the muscarinic receptor on MP generation, HUVEC and 16HBE cells were pretreated for 30 minutes with two different muscarinic antagonists (tiotropium and ipratropium) at various concentrations and then stimulated with ACh for 1 h as described above.

### Evaluation of MP generation

MP generation was investigated by different methods. The Zymuphen MP-activity kit measures the concentration of MP-associated PS based on the rate of prothrombin conversion to thrombin in an assay in which the availability of PS is the rate limiting step.

A cytofluorimetric analysis was conducted using a FACScanto**™**II flow cytometer (BD Biosciences, San Jose, CA, USA); MP were first discriminated by size, using calibration beads, as events conforming to a light scatter distribution within the 0.5–0.9 μm range in a SSc vs. FSc window and further identified as annexin V^+^ and CFSE^+^ events after incubation with allophycocyanin (APC)-annexin V and FITC-CFSE, in a APC vs. FITC window. Event acquisition was obtained at low flow rate and stopped after 210 s.

The number and dimension of MP were also assessed by nanoparticle tracking analysis (NTA) using a Nanosight NS300 NTA system (Malvern Panalytical Ltd., UK) which was equipped with a 488 nm laser and a sCMOS camera. For each sample, five videos of 30 sec were recorded at an automatically controlled temperature and the measurements were performed with constant sample flow of 70 using a syringe pump. The camera level was set to 14 and the detection threshold to 5. Collected data were analyzed with NTA 3.3 software.

### Measurement of [Ca^++^]_i_

16HBE or HUVEC (1.5 . 10^4^ cells/well) were loaded with 100 μL of the dye loading solution containing Fluo-4 NW dye and probenecid, according to the manufacturer’s instructions (Molecular Probes Fluo-4NWCalcium Assay kit)^18^. Briefly, the 96-well plate was incubated in the presence or absence of tiotropium at 37 °C for 45–60 min in the dark and ACh (1 mM) was added to the cells. The changes in Fluo-4 NW fluorescence were measured by the Wallac 1420 Victor 2 (Perkin–Elmer, Milan, Italy) at λ_ex_ 494 nm and λ_em_ 516 nm. Calcium mobilization was observed over time (up to 110 s) and analyzed by the Wallac 1420 Software version 3 (Perkin– Elmer Life and Analytical Sciences, Wallac, Milan, Italy). The increase in [Ca^2+^]_i_ fluorescence was expressed as relative fluorescence units (RFU).

### Evaluation of ERK phosphorylation

The involvement of ERK phosphorylation in ACh-induced MP generation was assessed by the FACE ERK 1/2 kit, a method that allows detection of protein phosphorylation directly in the cell, according to the manufacturer’s instructions.

### ELISA for chemokine detection

MP were incubated with HUVEC and 16HBE cells grown to confluence in 96-well plates for 24 h. Following an 18 h incubation, the conditioned medium was harvested, cleared by centrifugation for 5 min at 12,000xg and analyzed for IL-8 and MCP-1 content. IL-8 and MCP-1 in supernatants from HUVEC and 16HBE cells were measured by a sandwich ELISA kit (IL- 8 and MCP-1 Kit-Elisa-Ready-SET-Go!, Affimetrix, USA) with a microplate reader (iMark™ Microplate Absorbance Reader, Bio-Rad, Milan, Italy) according to the manufacturer’s instructions.

### Statistical analysis

Data are shown as mean ± SEM. Multiple comparisons were made with ANOVA for repeated measurements followed by Fisher’s least significant difference test using Prism software (GraphPad, San Diego, CA, USA). One-tail p values < 0.05 were considered statistically significant.
